# Clinical outcomes of curative surgical resection of peritoneal metastasis in patients with colorectal cancer: A long‐term follow‐up study

**DOI:** 10.1002/cam4.5195

**Published:** 2022-09-07

**Authors:** Kyunghye Bang, Jeong Eun Kim, Tae Won Kim, Sun Young Kim, Seok‐Byung Lim, In Ja Park, Chan Wook Kim, Yong Sik Yoon, Yong Sang Hong

**Affiliations:** ^1^ Department of Oncology, Asan Medical Center University of Ulsan College of Medicine Seoul Korea; ^2^ Division of Colon and Rectal Surgery, Department of Surgery, Asan Medical Center University of Ulsan College of Medicine Seoul Korea; ^3^ Division of Hemato‐Oncology, Department of Internal Medicine Chung‐Ang University Gwangmyeong Hospital Gwangmyeong Korea

**Keywords:** colorectal cancer, curative resection, peritoneal metastasis

## Abstract

**Introduction:**

Colorectal cancer with peritoneal metastasis (PM) has been considered a non‐curative disease. PM is associated with reduced overall survival (OS) and worse prognosis compared with metastasis at other sites. We aimed to investigate the treatment outcome and recurrence after curative resection of colorectal PM during a long‐term follow‐up.

**Methods:**

Patients who were diagnosed with colorectal PM and underwent surgery between December 2001 and December 2019 were included (*n* = 309). Curative resection was defined as PM resection without residual disease after surgery (complete macroscopic resection).

**Results:**

Of 309 patients, 208 (67.8%) had PM as an initially metastatic disease. Curative (R0/1) resection was achieved in 155 (50.2%) patients, while non‐curative operation (R2 resection or palliative operation including colostomy) was performed in 154 (49.8%) patients. Compared with patients who underwent non‐curative operation, those with curative resection more often had a single PM on preoperative imaging (34.2% vs. 20.8%, *p =* 0.011) and postoperative results (59.4% vs. 22.7%, *p* < 0.001) and less often had concurrent metastasis (distant lymph node, liver, or lung) at the time of surgery (*p* < 0.001). During a median follow‐up of 90.4 months, 80.6% (125/155) patients had recurrence in the curative resection group; the peritoneum was the most common site (56.0%). The median OS was 47.7 months (95% CI, 39.2–56.2) in the curative resection group and 24.8 months (95% CI, 20.8–28.9) in the non‐curative resection group, respectively (*p* < 0.001). In particular, twenty‐six patients without recurrence showed long‐term survival after curative resection (median OS, 87.1 months; range, 40.1–127.5).

**Conclusion:**

Surgical resection can be considered for selected patients with colorectal PM because a significant number of them could seize the cure changes during their treatment continuum.

## Graphical abstract

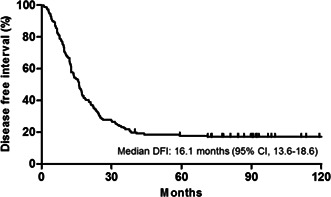

## 1 INTRODUCTION

Colorectal cancer (CRC) is the third most commonly diagnosed cancer and the second leading cause of cancer death worldwide.^1^ The treatment of CRC has evolved into a multimodal approach that includes surgery, radiotherapy or systemic chemotherapy, and local therapies to treat metastatic tumors regionally confined to specific organs such as the liver or lung, further improving the survival of patients with metastatic CRC.^2^, ^3^, ^4^

The peritoneum is the second most common metastatic site of CRC after the liver.^3^ Previous population‐based studies found synchronous and metachronous peritoneal metastasis (PM) in 8.3% of patients with CRC. The prevalence of synchronous PM was approximately 4–5%, and the cumulative incidence of metachronous PM was 4.2%. In 4.8% of the patients, the peritoneum was the only metastatic site at the time of diagnosis of metastatic disease.^5^, ^6^, ^7^

CRC with PM has long been considered a non‐curative disease, and PM is associated with reduced overall survival (OS) and worse prognosis compared with metastasis at other sites.^8^ Therefore, in recent years, multimodal strategies have been developed for the treatment of PM, especially cytoreductive surgery (CRS) with hyperthermic intraperitoneal chemotherapy (HIPEC), which was reported to improve survival (with a median OS of >40 months) in several previous studies.^9^, ^10^, ^11^ However, in a multicenter, randomized, phase III trial (PRODIGE 7), no significant difference in OS was found between the CRS plus HIPEC group (41.7 months; 95% confidence interval [CI], 36.2–53.8) and the CRS alone group (41.2 months; 95% CI, 35.1–49.7; *p* = 0.99) and postoperative late complications were more frequent in patients who underwent CRS plus HIPEC.^12^ These findings suggest that CRS alone could be the cornerstone of therapeutic strategies with curative intent for colorectal PM.

However, most studies on the surgical treatment of PM have been related to combination treatment with HIPEC^13^ or to peritoneal recurrence.^14^ Few reports have focused on surgical resection alone in patients with colorectal PM.^7^, ^15^, ^16^ Therefore, we aimed to investigate the treatment outcome and recurrence after curative resection of colorectal PM during a long‐term follow‐up in this study.

## 2 MATERIALS AND METHODS

### 2.1 Patients

This study was a retrospective analysis aiming to evaluate the clinical outcomes of surgical resection of PM in patients with CRC. Patients who were diagnosed with colorectal PM (i.e., initially metastatic disease, disease progression during treatment, recurrence after treatment, or incidentally discovered metastasis during surgery) and underwent surgery at Asan Medical Center, Korea, between December 2001 and December 2019, were identified. None of the patients included in this study underwent HIPEC.

The clinical and laboratory data of patients included in this study were retrospectively obtained by reviewing the medical records. This study was approved by the Institutional Review Board of Asan Medical Center (approval no. 2021–1441) and was performed in accordance with the ethical standards of the institutional research committee and the Declaration of Helsinki. The need for informed consent was waived because of the retrospective nature of the study.

### 2.2 Curative resection

Curative resection was defined as grossly complete resection of PM which included several types of surgery (colectomy combined with PM excision; local excision of PM; or PM excision combined with other metastasectomy such as bilateral salpingo‐oophorectomy, total abdominal hysterectomy, or partial hepatectomy). Patients with no microscopic residual disease (R0 resection) and those with minimal microscopic residual tumor (R1 resection)^17^ were considered to have had curative resection (complete macroscopic resection). Postoperative treatment was chosen with physicians' discretion.

### 2.3 Recurrence after curative resection

Recurrence after curative resection was diagnosed on follow‐up imaging, mainly with computed tomography (CT) and in some cases with fluorodeoxyglucose–positron emission tomography/CT as diagnostic support. The interval between curative resection and recurrence, the site of recurrence, and survival outcomes were reviewed.

### 2.4 Statistical analysis

Baseline patient characteristics are reported as counts and percentages and were compared using the χ^2^ test. Disease‐free interval (DFI) was defined as the time from the curative resection of PM to the date of tumor recurrence at any site. OS was defined as the time from the curative resection of PM to death of any cause. The cutoff date was July 31, 2021. DFI and OS were estimated using the Kaplan–Meier method and compared between the curative resection and non‐curative resection group using the Log‐rank test. A two‐sided *p*‐value of <0.05 was considered statistically significant. All statistical analyses were performed using Statistical Package for the Social Sciences (version 23.0; IBM).

## 3 RESULTS

### 3.1 Patient characteristics

**FIGURE 1:**
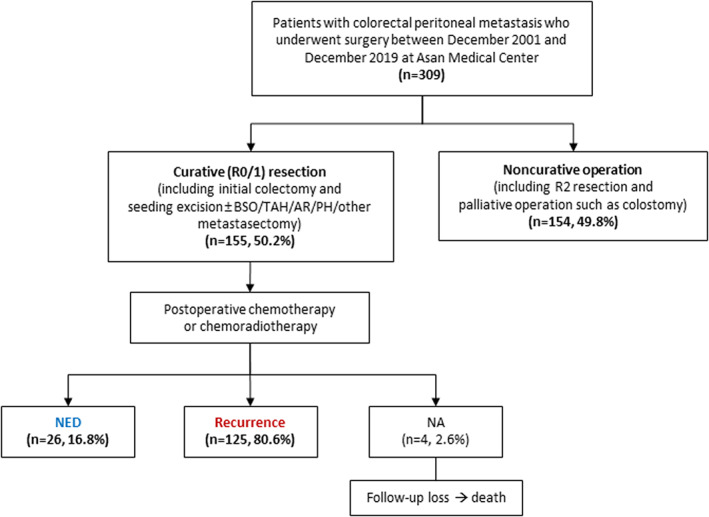
CONSORT diagram of patients. AR, anterior resection; BSO, bilateral salpingo‐oophorectomy; NA, not available; NED, no evidence of disease; PH, partial hepatectomy; TAH, total abdominal hysterectomy

Of 309 initially identified patients with colorectal PM who underwent surgery, 155 (50.2%) patients achieved curative (R0/1) resection, whereas 154 (49.8%) patients underwent non‐curative operation including R2 resection and palliative operation such as colostomy (Figure 1).

The baseline characteristics are summarized in Table 1. In the entire population (*n* = 309), the median age was 57 years (range, 17–83 years) and 57.0% (*n* = 176) were men. Most patients (97.7%) had an Eastern Cooperative Oncology Group performance status of 0 or 1 at the time of surgery. The non‐curative resection group had more patients with poorly differentiated tumor than the curative resection group (*p =* 0.006). No significant differences in mutations were observed between the two groups.

**TABLE 1:** Baseline characteristics

	Total		Curative (R0/1) resection		Noncurative (R2) resection		*p*‐value
	*N* = 309	%	*n* = 155	%	*n* = 154	%	
Age, years, median (range)	57 (17–83)						
Male sex	176	57.0%	84	54.2%	92	59.7%	0.325
ECOG performance status							0.709
0–1	302	97.7%	151	97.4%	151	98.1%	
2	7	2.3%	4	2.6%	3	1.9%	
Primary tumor site							**0.028**
Right colon	119	38.5%	48	31.0%	71	46.1%	
Left colon	133	43.0%	74	47.7%	59	38.3%	
Rectum	49	15.9%	30	19.4%	19	12.3%	
Multifocal	8	2.6%	3	1.9%	5	3.2%	
Histology							0.309
Adenocarcinoma	271	87.7%	140	90.3%	131	85.1%	
Mucinous carcinoma	32	10.4%	14	9.0%	18	11.7%	
Signet ring cell carcinoma	5	1.6%	1	0.6%	4	2.6%	
Tumor grade							**0.006**
W/D, M/D	242	78.3%	132	85.2%	110	71.4%	
P/D	34	11.0%	10	6.5%	24	15.6%	
Initial status of disease							**0.003**
Resectable disease	65	21.0%	44	28.4%	21	13.6%	
Locally advanced disease	13	4.2%	8	5.2%	5	3.2%	
Metastatic disease	231	74.8%	103	66.5%	128	83.1%	
*KRAS* mutation							0.215
Wild	141	45.6%	68	43.9%	73	47.4%	
Mutant	132	42.7%	64	41.3%	68	44.2%	
Unknown	36	11.7%	23	14.8%	13	8.4%	
*NRAS* mutation							0.615
Wild	132	42.7%	63	40.6%	69	44.8%	
Mutant	3	1.0%	1	0.6%	2	1.3%	
Unknown	174	56.3%	91	58.7%	83	53.9%	
*BRAF* mutation							0.093
Wild	246	79.6%	120	77.4%	126	81.8%	
Mutant	17	5.5%	6	3.9%	11	7.1%	
Unknown	46	14.9%	29	18.7%	17	11.0%	
MMR status							0.969
pMMR	274	88.7%	137	88.4%	137	89.0%	
dMMR	15	4.9%	8	5.2%	7	4.5%	
Unknown	20	6.5%	10	6.5%	10	6.5%	
MSI status							0.293
MSS	246	79.6%	124	80.0%	122	79.2%	
MSI‐L	13	4.2%	5	3.2%	8	5.2%	
MSI‐H	14	4.5%	10	6.5%	4	2.6%	
Unknown	36	11.7%	16	10.3%	20	13.0%	

Significant *p*‐values (< 0.05) are in bold.

Abbreviations: dMMR, deficient mismatch repair; ECOG, Eastern Cooperative Oncology Group; M/D, moderately differentiated; MMR, mismatch repair; MSI, microsatellite instability; MSI‐H, microsatellite instability‐high; MSI‐L, microsatellite instability‐low; MSS, microsatellite stable; P/D, poorly differentiated; pMMR, proficient mismatch repair; W/D, well differentiated.

### 3.2 Peritoneal metastasis

The characteristics of PM are summarized in Table 2. Of 309 patients, 208 (67.8%) were diagnosed with PM as an initially metastatic disease. On imaging at the time of PM diagnosis, a single PM was found in 85 (27.5%) patients and multiple (≥2) PMs were found in 89 (28.8%) patients. In 83 (26.9%) patients, no PM was detected on imaging examination but PM was found during surgery. Nonmeasurable metastatic lesions were also noted (*n* = 52, 16.8%), including peritoneal infiltration/thickening, pelvic ascites, and omental cake. The most common concurrent metastasis at the time of surgery was liver metastasis (*n* = 78, 25.2%), followed by distant lymph node (*n* = 50, 16.2%) and lung (*n* = 31, 10.0%) metastases.

**TABLE 2:** Characteristics of peritoneal metastasis

	Total		Curative (R0/1) resection		Noncurative (R2) resection		
	*N* = 309	%	*n* = 155	%	*n* = 154	%	*p*‐value
PM status							**<0.001**
Initially metastatic disease	208	67.3%	91	58.7%	117	76.0%	
Disease progression	31	10.0%	14	9.0%	17	11.0%	
Recurrence	70	22.7%	50	32.3%	20	13.0%	
No. of PMs on imaging at the time of PM diagnosis							**0.011**
None	83	26.9%	42	27.1%	41	26.6%	
Single	85	27.5%	53	34.2%	32	20.8%	
Multiple (≥2)	89	28.8%	33	21.3%	56	36.4%	
Nonmeasurable^a^	52	16.8%	27	17.4%	25	16.2%	
Concurrent metastasis at the time of surgery							
Lymph node (M1)	50	16.2%	11	7.1%	39	25.3%	**<0.001**
Liver	78	25.2%	24	15.5%	54	35.1%	**<0.001**
Lung	31	10.0%	5	3.2%	26	16.9%	**<0.001**
Ovary	26	8.4%	11	7.1%	15	9.7%	0403
Others	7	2.3%	2	1.3%	5	3.2%	0.248
Type of surgery							**<0.001**
PM excision only	45	14.6%	38	24.5%	7	4.5%	
PM excision with resection of other tumors or organs	142	46.0%	108	69.7%	134	87.0%	
PM excision with ileostomy or colostomy due to obstruction	16	5.2%	5	3.2%	11	7.1%	
Peritonectomy/omentectomy	6	1.9%	4	2.6%	2	1.3%	
No. of PMs in surgery notes and pathology reports							**<0.001**
Single	127	41.1%	92	59.4%	35	22.7%	
Multiple (≥2)	182	58.9%	63	40.6%	119	77.3%	

Significant *p*‐values (< 0.05) are in bold.

Abbreviation: PM, peritoneal metastasis.

^a^ Nonmeasurable lesions include peritoneal thickening, peritoneal infiltration with ascites, and omental cake.

The initial diagnosis of PM as a recurrence was more common in patients with curative resection than in those with non‐curative operation (32.3% vs. 13.0%, *p <* 0.001). The proportion of patients with a single PM on both the initial diagnostic imaging of PM and the postoperative results (*p =* 0.011 and *p* < 0.001) was significantly higher in the curative resection group than in the non‐curative operation group. Patients with curative resection less frequently had concurrent metastasis (distant lymph node, liver, or lung metastasis) at the time of surgery than those with non‐curative operation (*p <* 0.001).

### 3.3 Recurrence after curative resection and survival

After resection of PM, 288 (93.2%) patients were treated with post‐operative chemotherapy. Of the remaining 21 patients, eight (2.6%) patients received post‐operative radiotherapy, and twelve (3.9%) patients underwent active surveillance.

**FIGURE 2:**
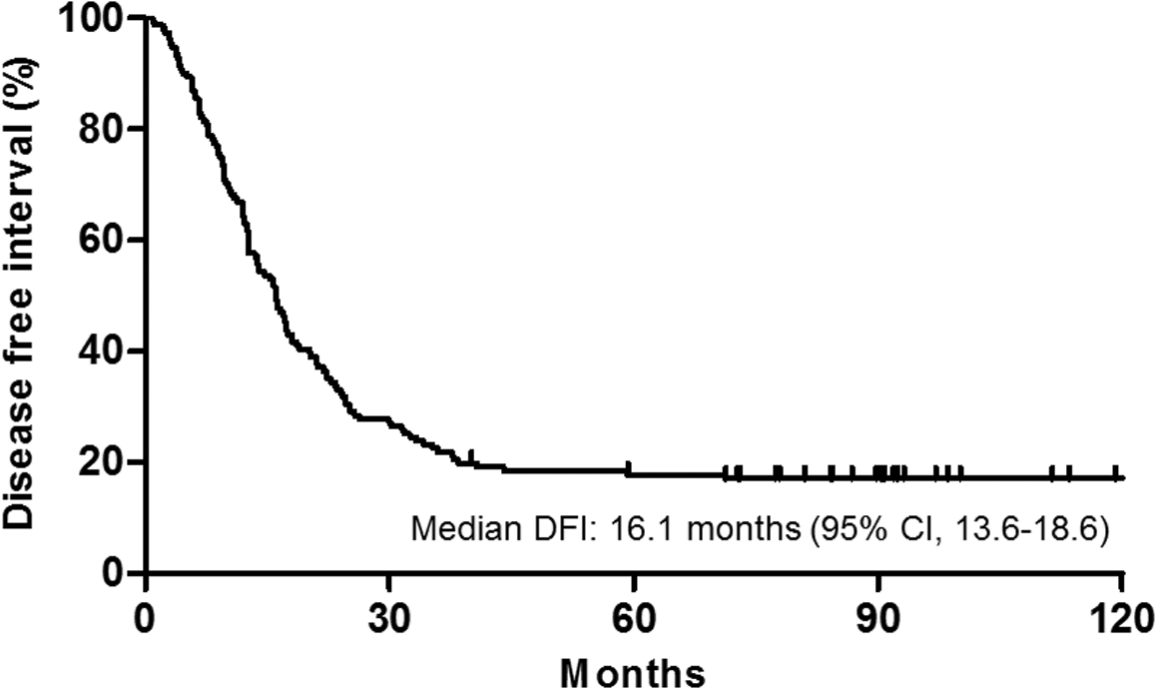
Disease‐free interval after curative resection of peritoneal metastasis from a colorectal origin. CI, confidence interval; DFI, disease‐free interval

In 155 patients with curative resection, during a median follow‐up of 90.4 months (7.5 years), 125 (80.6%) recurrence events occurred (Table 3). The most common site of recurrence was the peritoneum (56.0%). The median DFI was 16.1 months (95% CI, 13.6–18.6 months) (Figure 2). The 6‐month and 1‐year disease‐free rates were 86.8% and 64.9%, respectively. In univariate analysis, a single PM (vs. ≥2) was significantly associated with lower recurrence after surgery (hazard ratio [HR], 0.58; 95% CI, 0.39–0.85; *p* = 0.006) and the absence of concurrent metastasis tended to reduce recurrence (HR, 0.70; 95% CI, 0.47–1.04; *p* = 0.081).

**TABLE 3:** Recurrence after curative resection

	Curative (R0/1) resection	
	*n* = 155	%
Disease‐free interval, months, median (95% CI)	16.1 (13.6–18.6)	
Recurrence events	125	80.6%
Recurrence sites		
Lymph node	19	15.2%
Liver	32	25.6%
Lung	34	27.2%
Peritoneum	70	56.0%
Ovary	3	2.4%
Others	15	12.0%
Overall survival in patients with NED (*n* = 26), years, median (range)	7.4 (3.3–10.6)	

Abbreviations: CI, confidence interval; NED, no evidence of disease; PM, peritoneal metastasis.

The median OS of patients with curative resection was 47.7 months (4.0 years) (95% CI, 39.2–56.2 months) However, the median OS of patients with non‐curative resection was 24.8 months (2.1 years) (95% CI, 20.8–28.9 months) (*p* < 0.001) (Figure S1).

Interestingly, among patients with curative resection, a total of 26 (16.8%) patients still had no evidence of disease (NED) at the last follow‐up examination and showed long‐term survival after curative resection, with a median OS of 87.1 months (7.4 years) (range, 40.1–127.5 months; 95% CI, not assessable) (Figure S2). One patient (3.8%) died of sudden cardiac arrest that was not related to tumor recurrence.

## 4 DISCUSSION

This retrospective analysis of patients with CRC with PM who underwent surgery demonstrated that patients with curative (R0/1) resection accounted for one‐half of the total patients and more frequently had a single PM and less frequently had concurrent metastasis than patients with non‐curative operation (R2 resection or other palliative operation). Among patients with curative resection, recurrence occurred in 80.6% and the most common site of recurrence was the peritoneum. However, in patients who did not have recurrence, the NED status was maintained for a long time despite previously having PM.

PM has been known as a predictor of worse prognosis in patients with metastatic CRC, on its own or compared with metastases in other sites (OS, 16.3 months for PM, 19.1 months for liver‐only metastasis, and 24.6 months for lung‐only metastasis).^6^, ^8^, ^18^ However, several previous studies have reported that R0 to R1 resection contributes to better outcomes in patients with PM from CRC.^15^, ^16^, ^19^ These findings suggest that curative resection of PM could improve the prognosis of patients with PM comparable to that of patients with CRC with metastasis at other sites. The present study also showed this improvement in survival outcome in patients with curative resection of PM.

To further improve the efficacy of surgical resection of colorectal PM, HIPEC has been added to CRS and the combination treatment produced better survival outcomes in several retrospective studies.^9^, ^10^, ^11^ However, in a phase 3, prospective trial (PRODIGE 7), there was no survival difference between the CRS with HIPEC group and the surgery alone group.^12^ In addition, CRS with a curative intent is a good choice for patients with a low burden of metastases and favorable tumor biology in a recently updated US guidelines (The Chicago Consensus).^20^ Therefore, in light of the results of the previous studies and those of our study, we believe that surgery alone can also be considered a therapeutic option in patients with colorectal PM.

However, even after curative resection of PM, >70% patients relapsed and the most common site of recurrence was still the peritoneum,^21^, ^22^ which differs from previous reports that the liver was the most common recurrence site regardless of the presence or site of initial metastasis.^23^ To reduce recurrence, adjuvant chemotherapy after metastasectomy for liver or lung metastasis has been used^24^, ^25^, ^26^; however, there is still little evidence on its effect on PM. In a previous study, in patients with isolated synchronous colorectal PM who underwent upfront surgery, adjuvant systemic chemotherapy seemed to be associated with improved OS.^13^ Eventually, if systemic chemotherapy is required after surgery, it is important to select patients who are expected to obtain greater benefits from surgery for metastatic CRC in the first place, and our study may be helpful in this regard. The chemotherapy regimen after surgery differs across institutions, and a recent study showed that targeted therapy produced better OS than cytotoxic chemotherapy.^16^ In the current study, most common perioperative chemotherapy was 5‐fluorouracil/leucovorin combined with irinotecan (FOLFIRI) (*n* = 69, 22.3%), followed by bevacizumab plus FOLFIRI (*n* = 59, 19.1%) and 5‐fluouracil/leucovorin combined with oxaliplatin (FOLFOX) (*n* = 58, 18.8%). Although direct comparison is difficult due to various setting of patients, there was no significant difference in survival outcome with or without targeted therapy (*p* = 0.367). Therefore, the most appropriate regimen for PM from CRC needs to be clarified in the future.

The median OS in patients with NED in the curative resection group indicated long‐term survival, and a single PM and minimal or no other concurrent metastatic lesions were associated with lower recurrence. According to previous studies, a smaller extent of PM is associated with a better prognosis^7^, ^10^, ^19^, ^27^ and macroscopic radical resection including R0 resection per se was also an independent predictor of good prognosis.^7^, ^27^, ^28^ Therefore, selecting patients with a single PM and no concurrent metastasis and performing resection with curative intent could improve the survival outcomes in metastatic CRC.

Our study had several limitations. First, this study may be limited by its retrospective design, which is subject to unintentional biases and lack of clinical information such as surgical complications and toxicity. Further, it was difficult to collect all CEA levels evenly and interpret them because the patients had different settings and this study was retrospective. Second, a single PM was more common in the curative resection group. This may be because surgical resection with curative intent was more frequently attempted for a localized solitary PM, which might have introduced a selection bias. Third, the settings of patients, including disease status and operation type, were heterogeneous. It could lead to a selection bias and seems difficult to generalize the data. However, this also shows that if complete macroscopic resection is possible for PM, curative resection seems an acceptable treatment option in any setting of PM. Last, we did not use the peritoneal cancer index (PCI), a sum of the scores of lesion size in 13 abdominal regions, which has been used in previous studies on colorectal PM.^29^, ^30^ Although the PCI is a prognostic factor in patients with PM from CRC, it is difficult to apply to every operation because it needs to be assessed by dividing the regions and requires the surgeon to count and measure each lesion during the operation. Therefore, the current study is clinically meaningful because it shows that surgical resection with curative intent is feasible for an appropriately selected population with only a single colorectal PM without or with minimal concurrent metastases; thus, better prognosis could be expected without assessing the PCI in these patients. Furthermore, our results could help increase the number of candidates for curative resection of colorectal PM.

## 5 CONCLUSION

In summary, around 20% of patients with colorectal PM could be cured after curative intent surgical resection. Selection of surgical candidates needs careful consideration because the recurrence rate still remains high.

## AUTHOR CONTRIBUTIONS

Study concept: YS Hong, JE Kim; study design: YS Hong, JE Kim; data acquisition: all authors; data analysis and interpretation: JE Kim, KH Bang; statistical analysis: KH Bang; manuscript preparation: JE Kim, KH Bang; manuscript editing: YS Hong, JE Kim, KH Bang; manuscript review and approval: all authors.

## FUNDING INFORMATION

This research received no specific grant from any public, commercial, or not‐for‐profit funding agency.

## CONFLICT OF INTEREST

The authors declare that they have no competing interests.

## ETHICS STATEMENTS

This study was approved by the Institutional Review Board of Asan Medical Center (approval no. 2021–1441) and was performed in accordance with the ethical standards of the institutional research committee and the Declaration of Helsinki. The need for informed consent in this study was waived, as Korean regulations do not require consent for retrospective analyses.

## Supporting information


Figure S1
Figure S2

## Data Availability

The data that support the findings of this study are available from the corresponding author.
