# Supplementary figures and images for: Clinical outcomes of curative surgical resection of peritoneal metastasis in patients with colorectal cancer: A long‐term follow‐up study
Source: Cancer Med. 2022 Sep 7;12(3):2861–8. doi: 10.1002/cam4.5195 (PMC9939134; doi:10.1002/cam4.5195)

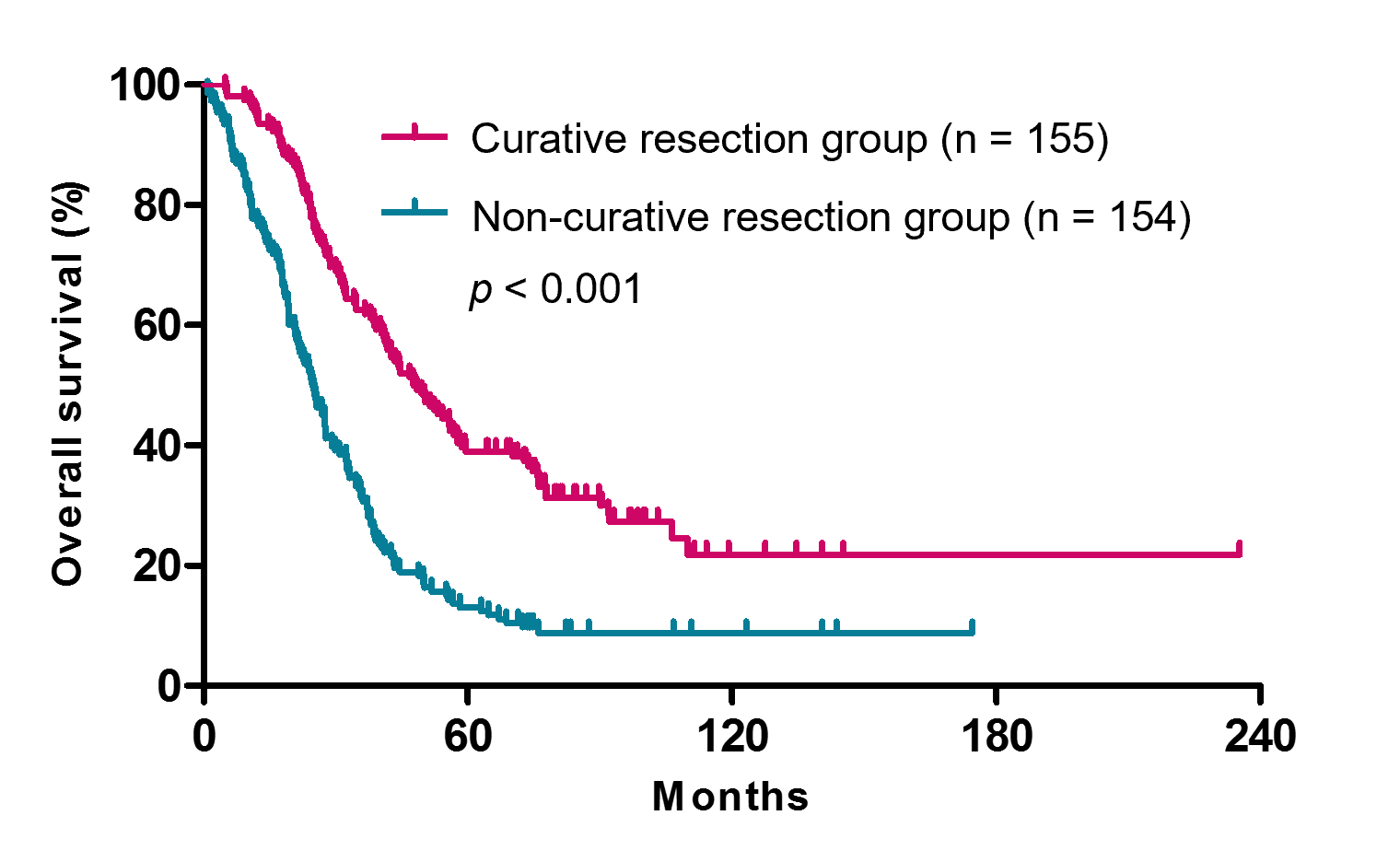

Supplement: Supplementary file 1 — Figure S1 Figure S2 [file CAM4-12-2861-s001.zip › CAM4_5195_Supplementary Figure 1A (edited).tif]

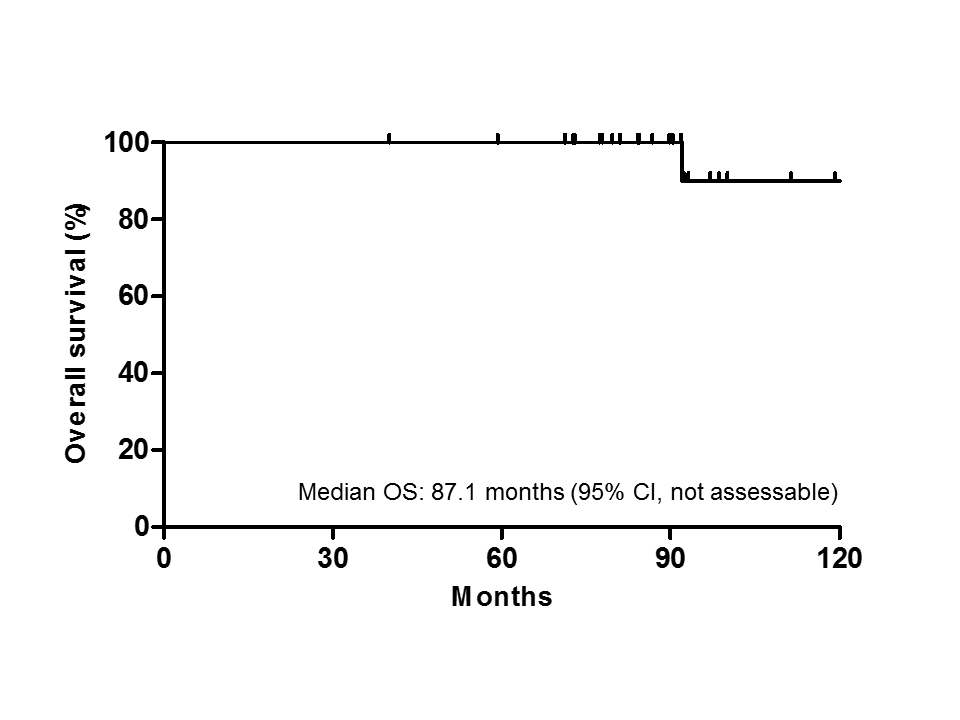

Supplement: Supplementary file 1 — Figure S1 Figure S2 [file CAM4-12-2861-s001.zip › CAM4_5195_Supplementary_Figure_1B.TIF]
